# Mid infrared spectroscopy combined with chemometrics as tool to monitor the impact of heat stress and dietary interventions in lactating sows

**DOI:** 10.1007/s00484-024-02792-5

**Published:** 2024-10-26

**Authors:** M. Navarro, A. Coba, M. Muller, E. Roura, D. Cozzolino

**Affiliations:** 1 Centre for Nutrition and Food Sciences, Queensland Alliance for Agriculture and Food Innovation (QAAFI), The University of Queensland, Brisbane, QLD 4072 Australia; 2 Centre for Animal Science, QAAFI, The Univeristy of Queensland, Brisbane, QLD 4072 Australia

**Keywords:** Lactating sow, Heat stress, Infrared, High throughput, Biomarkers

## Abstract

Heat stress in hyper-prolific lactating sows is recognised as a factor reducing feed intake, milk production, and welfare, with significant losses in farm productivity. Individual capacities for body thermoregulation during environmental hyperthermia determine the adaptation of the animal during long and recurrent events. This study aimed to evaluate the ability of attenuated total reflectance (ATR) mid infrared (MIR) spectroscopy as a high-throughput method to identify markers of stress in plasma and milk collected from lactating sows under heat stress conditions fed with two levels of protein in the diet defined as low (16%) and standard (20%). The MIR spectra were analysed using linear discriminant analysis (LDA) and principal component analysis and validated using cross-validation. The results obtained indicated that MIR spectroscopy, in combination with chemometrics, was able to identify changes in the spectra associated with heat stress in wavenumbers corresponding with amide groups (proteins) (highest loadings observed in the regions between1065 and 1635 cm^−1^), lipids and unsaturated fatty acids (regions between 1746 and 3063 cm^−1^), lipo-polysaccharides (in 1247 cm^−1^) and carbohydrates (around the region1050 cm^−1^). These results also indicated that the information provided by these wavenumbers can be used as metabolic markers of the adaptation of the sows to hyperthermia. It was concluded that MIR spectroscopy is a rapid and inexpensive tool capable of detecting and evaluating the main biochemical changes of hyperthermia on lactating sows, facilitating the development of palliative management strategies such as dietary manipulations.

## Introduction

A heat-tolerant animal is defined as one that can maintain homeothermy under high environmental heat loads (Abdelnour et al. 2019). The maintenance of homeothermy relies on the animal's capacity to balance thermogenesis and heat dissipation (Sejian et al. 2018). Various methods and techniques have been proposed to identify heat-sensitive animals and monitor the consequences of heat stress, including measuring skin and core body temperature, respiration rate (RR), and heart rate (HR) (Abdelnour et al. 2019; Sejian et al. 2018). Recent reports have also indicated that heat stress responses can be measured using specific biomarkers present in saliva, plasma, or milk (Tian et al. 2015, 2016). Advances in omics techniques and high-throughput methods, combined with multivariate data tools (e.g. chemometrics and machine learning), have provided with new analytical possibilities to measure biomarkers and to better understand the mechanisms of heat stress in livestock breeding programs in intensive production systems (Loor et al. 2013; Carabaño et al. 2017; Koltes et al. 2018; Abdelnour et al. 2019; Sejian et al. 2018).

Infrared (IR) spectroscopy has been employed as an analytical method in nutrition and biomedical research and has been applied as a diagnostic tool for measuring a wide range of biomarkers in different biological fluids and tissues (e.g., skin, plasma, saliva, and milk) (Khaustova et al. 2010; Lin et al. 2007; Naseer et al. 2020; Naumann 2011; Orphanou et al. 2015). The basic principle of IR spectroscopy involves using light to measure molecular vibrations of the different bonds between atoms (carbon, nitrogen, oxygen, and hydrogen) present in a sample (e.g. tissue, animal fluids) (Shaw and Mantsch 1999; Lin et al. 2007; Naumann 2011; Movasaghi et al. 2008; Ellis and Goodacre 2006). Particularly, the mid-infrared (MIR) region is characterized by the analysis of the fundamental vibrational modes of the molecules within the spectral range of 4000 – 400 cm^−1^. The narrow region between 1800 – 900 cm^−1^ is known as the fingerprint region (Shaw and Mantsch 1999; Elkins 2011; Ellis and Goodacre 2006; Movasaghi et al. 2008). This region provides unique information about the fundamental vibrations of molecules or biomarkers presents in biological samples, including amide groups of proteins, as well as lipids, esters, fatty acids, and carbohydrates (Ellis and Goodacre 2006; Movasaghi et al. 2008).

The identification and quantification of biomarkers associated with heat stress using MIR spectroscopy have been reported in dairy cattle, primarily using milk as the preferred sample matrix (Grelet et al. 2023; König and May 2019; Carabaño et al. 2017; Hammami et al. 2015; Min et al. 2017; Tian et al. 2015, 2016). In these studies, the MIR spectrum of the milk samples was used to develop calibration models to quantify the level of specific metabolites or biomarkers associated with heat stress (Carabaño et al. 2017; Hammami et al. 2015; Min et al. 2017). Hammami and collaborators (2015) also explored the use of MIR spectroscopy to quantify the concentration of fatty acids in milk as potential biomarkers to study the effects of heat stress in dairy cattle. However, no reports have been found in the scientific literature on using MIR spectroscopy to monitor heat stress in lactating sows.

Developments and applications of IR techniques have been made possible by the utilization of chemometrics and machine learning methods or techniques (Karoui et al. 2010; Bureau et al. 2019). These techniques are utilised to analyse and interpret the inherent complexity of the IR spectra, to develop classification models or to develop quantitative models (e.g. calibration) (Karoui et al. 2010; Bureau et al. 2019). Among these techniques, principal component analysis (PCA), discriminant techniques such as linear discriminant analysis (LDA), cluster analysis, artificial neural networks, (ANN) and support vector machines (SVM) are the most commonly used and reported in the scientific literature (Bureau et al. 2019).

Nutritional approaches, such as reduced dietary protein, to ameliorate animal hyperthermia, are also under scrutiny. Undigested protein from feed reaching the large intestine is available for proteolytic fermentation by the microflora, leading to the accumulation of end products and the production of metabolic heat (Gilbert et al. 2018). Reduced levels of protein in the diet could potentially decrease proteolytic fermentation and metabolic heat production (Zhang et al. 2020). For modern sows, lactation is a very sensible period to the effects of heat stress where intense milk production to feed large litters is linked to a greater metabolic rate, increasing the internal head load of the animal (Zhang et al. 2020).

This study aimed to evaluate the ability of attenuated total reflectance (ATR) MIR spectroscopy as a high-throughput method to analyse plasma and milk collected from lactating sows under heat stress conditions fed a diet with two protein levels: low (16%) and standard (20%).

## Materials and methods

### Animals and housing

The animal trial involved 16 multiparous Large White sows (273.25 ± 6.47 kg body weight, body condition 3.4 ± 0.2, parities 4.25 ± 0.45) selected from the UQ Piggery at the University of Queensland, Gatton campus. Sows were housed in one of two twin climate-controlled rooms in the Queensland Animal Science Precinct (QASP) one week before farrowing. Each room had two parallel rows of four farrowing pens (2.1 × 2.1 m each) equipped with a fully slatted plastic floor, two independent drinkers (sow and piglets), and a creep area with a mat floor and a heating lamp. The experimental period included one week of adaptation to the rooms before farrowing. Both rooms maintained a steady environmental temperature of 20 °C until farrowing. Farrowing was induced via Lutalyse® injection (1 mL) the day before the due date, at 8:00 AM and 15:00 PM.

### Experimental design, feed and environmental conditions

The day after farrowing, and during all the lactation (30 days), sows experienced four thermic periods, each consisting of 3 days of environmental heat (HS) and 3 days of thermoneutral (TN) conditions, with a 2-day washout period (for dietary purposes) at TN conditions (Table 1). Humidity was maintained at approximately 60% (61.6 ± 0.18%) in both rooms throughout the experiment. The HS program involved a baseline temperature of 20 °C Heat Index Temperature (HIT) of 20 °C and 2 °C increments every hour starting at 8:00 am until reaching 33 °C at 12:30 PM (HIT 40 °C). The maximum temperature was then maintained for 2 h before being reduced by 2 °C every hour until returning to baseline at 20 °C. This daily cycle was repeated for 3 days in each period. The first day of HS in each period was acute HS (AHS), and the last (third) day was chronic HS (CHS) stress, where adaptation mechanisms may be activated by the heatwave (Table 1).

**Table 1:**
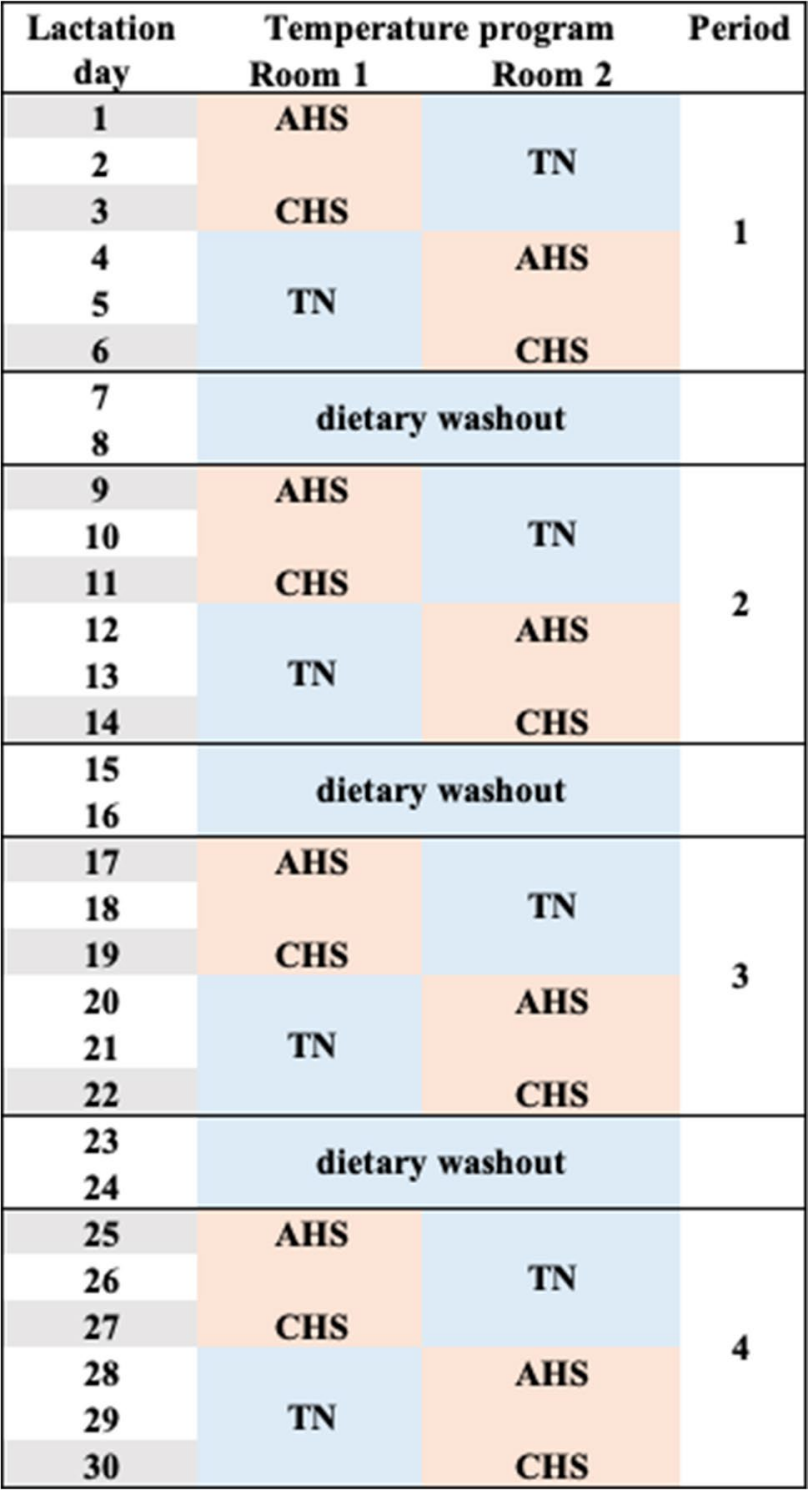
Experimental design including the daily temperature program in both climate-controlled rooms (1 and 2) during the trial. The lactation period was divided in 4 period of six days, including 3 days of heat stress (HS) and 3 days of thermoneutral environment (TN). Beige colour background refer to HS and blue colour background refers to TN. Acute HS (AHS) refer to the first day of heat stress in each thermal period, while Chronic HS (CHS) refers to the last day of HS in each period. Days with grey background refer to the day that blood and milk samples were collected in each period

During TN periods, one room maintained a steady environmental temperature of 20 °C, while the other operated in the opposite environmental condition. The HS-TN/TN-HS cycle was repeated four times (periods) in each room, ensuring that at any given time, one room (8 sows) had TN conditions and the other room (8 sows) had HS conditions. Each sow underwent four periods of HS and four periods of TN throughout lactation.

During each 6-day thermic period (3 days under TN and 3 days under HS), each sow received either a standard lactation diet with 20% crude protein (CP) or an experimental low-protein diet with 16% CP (Table 2). Both diets were based on wheat and iso-caloric principles (14 MJ/kg Digestible Energy for both diets). Between two thermic periods, sows received a washout feed with 17.5% CP and 14 MJ/kg in TN conditions for 2 days. By the end of the experiment, each sow in each temperature condition (HS and TN) received each dietary treatment twice, following a Latin Square Design with a factorial design of 2 dietary treatments and 2 environmental temperatures, resulting in 64 replicates for each treatment and temperature event (sows were used as paired control). The 2-day washout period after each period aimed to clear the gastrointestinal tract and the circulatory system of the previous feed, avoiding potential carryover effects. Daily feed intake was collected and expressed as average daily feed intake (ADFI).

**Table 2:** Composition of Standard (19.19%, SP) and Low (14.08%, LP) protein diets for sows. Washout feed was formulated as 50% SP and 50% LP feed

Ingredients, %	Standard Protein	Low Protein
Wheat	65.53	73.39
Barley	10.00	10.06
Canola meal	5.00	-
Soyabean meal	9.35	-
Soyabean full fat	5.40	3.77
Soycomil^1^	-	2.01
Canola oil	1.00	1.51
Dextrose	-	4.02
Limestone	1.10	1.16
Monodicalcium phosphate	1.35	1.66
Salt	0.30	0.30
Choline chloride	-	0.14
Lysine HCL	0.41	0.73
DL-Methionine	-	0.12
Threonine	0.11	0.28
Tryptophan	-	0.05
L-Valine	0.12	0.30
L-Isoleucine	-	0.17
Mycosorb^2^	0.05	0.05
Bentonite	0.08	0.08
Vitamin & mineral premix^3^	0.2	0.2
Nutrients (%)	Standard Protein	Low Protein
Dry matter	89.74	90.28
Digestible energy	13.99	14.08
Protein	19.19	15.68
Crude Fibre	3.00	2.20
Fat	3.80	3.90
IIe	0.73	0.71
Val	0.98	0.95
Lys	1.13	1.09
Met	0.30	0.35
Thr	0.76	0.73
Trp	0.26	0.25
AILYSPIG^4^	0.99	1.01
M + C	0.67	0.65
Calcium	0.75	0.76
Phosphorus	0.67	0.66
Available phosphorus	0.40	0.45
Analysed composition	%	%
Moisture	7.64	7.41
Ash	5.20	6.44
Neutral detergent fibre	58.03	46.27
Crude protein	18.26	16.40
Crude fat	2.64	2.71

^1^Soycomil: high-quality soy protein concentrate; ^2^ Mycosorb: mycotoxin binder; ^3^ Premix provided per kilogram of diet (as-fed basis): VA, 10000 IU; VD_3_, 1800 IU; VE, 100 IU; Biotin, 0.3 mg; Folic, 2.5 mg; Choline, 1401.49 mg; VB_12_, 0.04 mg; Iron, 96 mg; Copper, 8.0 mg; Zinc, 120 mg; Manganese, 40 mg; Iodine, 0.56 mg; Selenium, 0.4 mg ^4^ AILYSPIG: available ileal Lys

### Sampling and measurements

Physiological parameters were recorded during TN and HS conditions on days 1 and 3 at 12:30 PM in every thermal period. Core body temperature was continuously measured using an iButton datalogger (iButton DS1921-GF5, temperature range -40 to 85 °C, accuracy of 11-bit for ± 0.00625 °C resolution, RS Components, Australia) loaded into a blank CIDR intravaginal device implanted in the sow's vagina. The datalogger recorded temperature at 15-min intervals. Diurnal vaginal temperature (VT) was the average of datalogger records from 12 to 1 PM, while nocturnal VT was the average from records between 11 PM to 12 AM. Rectal temperature was measured daily at 1 PM using a digital thermometer (Omron MC-343, Singapore) with an accuracy of 0.1 °C. Respiratory rate (RR) was measured as breaths per minute using a stopwatch simultaneously (1 pm). Blood and milk samples were obtained from all sows during TN (third day) and HS on the first (AHS) and third (CHS) days of each period. Blood samples were collected using a BD® EDTA 10 mL vacutainer tube from the mammary vein (Scollo et al. 2019). Milk sample collection involved separating the lactating pigs from the sow for one hour. One hour after the piglets were separated from the dam, the udder was cleaned using a disinfectant wipe, with a gentle massage to stimulate milk ejection. For the milk collection, the operator pressed the nipple gently from the top to the tip. Approximately 5 mL of sow milk was collected from each sow into a plastic sterile tube. Blood and milk samples were kept on ice until for 30 min maximum before centrifugation. Samples were centrifugated at 4 °C during 10 min at 3500 rpm., aliquoted in 2 mL Eppendorf tubes and stored at -80 °C until analysis.

### Infrared spectra of milk and plasma

The MIR spectrum of milk and plasma samples from each animal was obtained using an ALPHA II (Bruker Optics, Ettilgen, Germany) (4000 cm^−1^ to 400 cm^−1^ region) equipped with a diamond attenuated total reflection (ATR) crystal. Milk and plasma samples were thawed at room temperature (25 °C) before MIR measurement. The ATR crystal was fully covered with either the mil or plasma sample (approx. 5 µL) where the spectrum of each sample was immediately recorded. Furthermore, each spectrum was computed using an average of 24 coadded interferograms at a resolution of 4 cm^−1^. The spectrum of air served as background before sample measurement, and the spectrum of water was measured every 20 samples and used as quality control. The instrument was operated using the OPUS software (version 8.5.29, Bruker Optics, Ettilgen, Germany). After each measurement, the surface of the ATR crystal was cleaned using a 70% w/w ethanol–water solution and dried with tissue paper between samples. In total, the spectra of 317 samples (162 plasma and 155 milk samples) were collected.

### Chemometric and statistical analysis

Before chemometric analysis, the MIR data of both of milk and plasma samples were pre-processed using baseline correction and Savitzky-Golay second derivative (second polynomial order and 21 smoothing points) (The Unscrambler X, Camo, Oslo, Norway) (Savitzky and Golay 1964). Principal component analysis (PCA) and linear discriminant analysis (LDA) were developed using full cross-validation (leave one out) (Bureau et al. 2019). Both milk and plasma samples from the animals under AHS and CHS were grouped together and labelled as "heat stress" to develop the LDA models. The percentage of correct classification was used to evaluate the accuracy of the LDA models. Additionally, the absorbances at specific wavenumbers (e.g. amide, lipids, carbohydrates) were statistically analysed, and means compared using a *t*-student test (*P* < *0.05*). Differential spectrum analysis was applied by subtracting the averaged spectra of AHS and CHS blood sample groups from TN groups and CHS from AHS groups in each diet.

The effect of heat stress on sows' feed intake and physiological parameters was analysed using a *t*-student test and plotted using Graphpad Prism 10 (GraphPad Software, California, USA). The number of samples (n) refers to the number of pigs used. Following animal welfare principles, 2 sows needed to be removed from the trial, resulting in a total of *n* = 14 sows used (included in the statistical analysis). Results were considered statistically significant at *P* < *0.05*.

## Results

### Feed intake and physiological parameters response

**Fig. 1:**
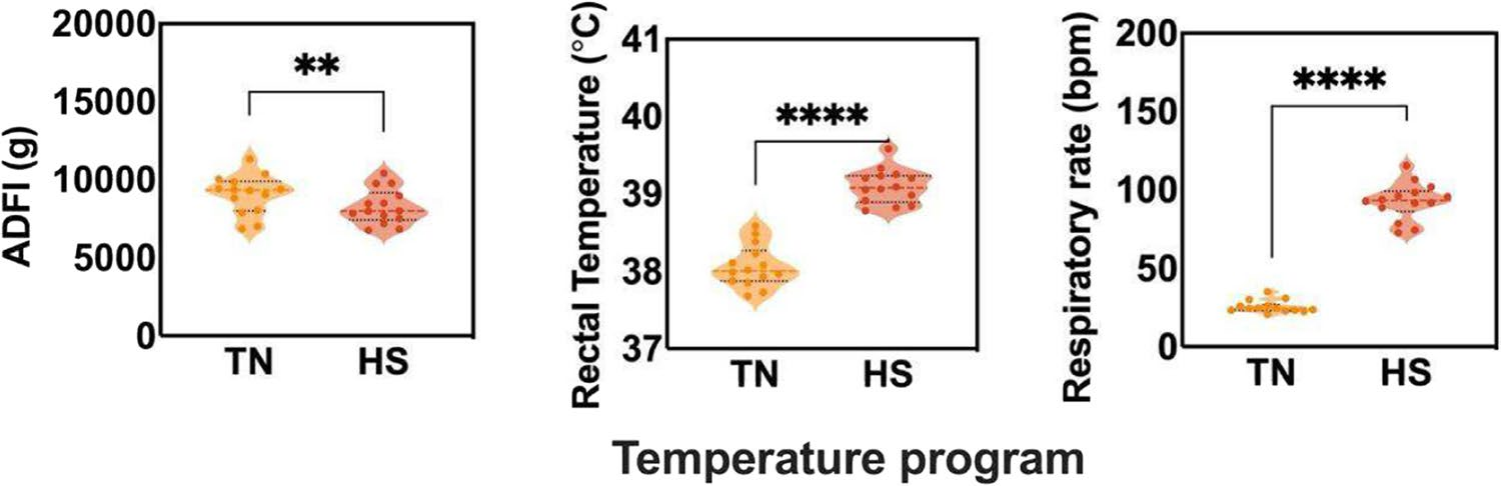
Changes in feed intake, rectal temperature and respiratory rate comparing the sows under thermoneutral or heat stress conditions (*n* = 14 sows). Average Daily Feed Intake (ADFI, g), Rectal Temperature (RT, °C) and Respiratory Rate (RR, breaths per minute) were measured under thermoneutral (TN) or heat stress (HS) environments. RT and RR data were collected at the same time of the day, at peak temperature during the HS period (1 pm). The line in the graphs indicates mean ± standard error. Dot points show the distribution of the individual values and * indicate statistically significant differences (*:*P* < 0.05,**: *P* < 0.01,****:*P* < 0.0001)

**Fig. 2:**
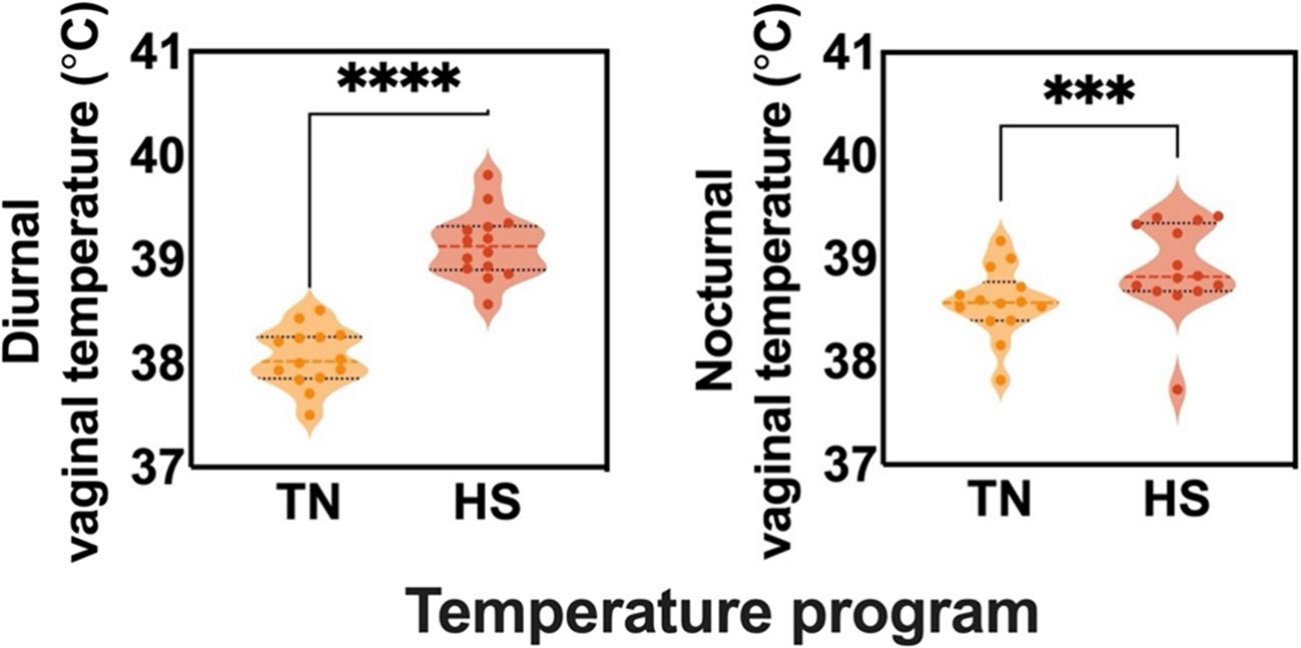
Diurnal and nocturnal core temperature measured in the vagina using a. Average of the vaginal temperature (VT) during the environmental temperature peak (12–1 pm), referred to as “Diurnal”, compared to the low environmental temperature at night (11 pm-12 am), referred to as “Nocturnal” using an internal probe in the lactating sows. The line in the graphs indicates mean ± standard error. Dot points show the distribution of the individual values and * indicate statistically significant differences (*:*P* < 0.05,**: *P* < 0.01,****:*P* < 0.0001)

At the end of the lactation period, on average, the final body weight of the sows was 256 ± 19.43 kg, while the final body condition of the animals was, on average, 3.2 ± 0.5. During HS conditions, the average daily feed intake (ADFI) significantly decreased (*P* = *0.002*) compared to the TN period (see Fig. 1). Respiratory rate (RR) increased significantly (*P* < *0.001*) in sows during acute HS (AHS) and chronic HS (CHS) stress, indicating characteristic panting behaviour (Fig. 1). Both rectal temperature (RT) (Fig. 1) and internal body temperature (VT) (Fig. 2) increased significantly (*P* < *0.0001* and *P* = *0.0002*, respectively) during HS compared to TN.

### Interpretation of the MIR spectra of plasma samples

**Fig. 3:**
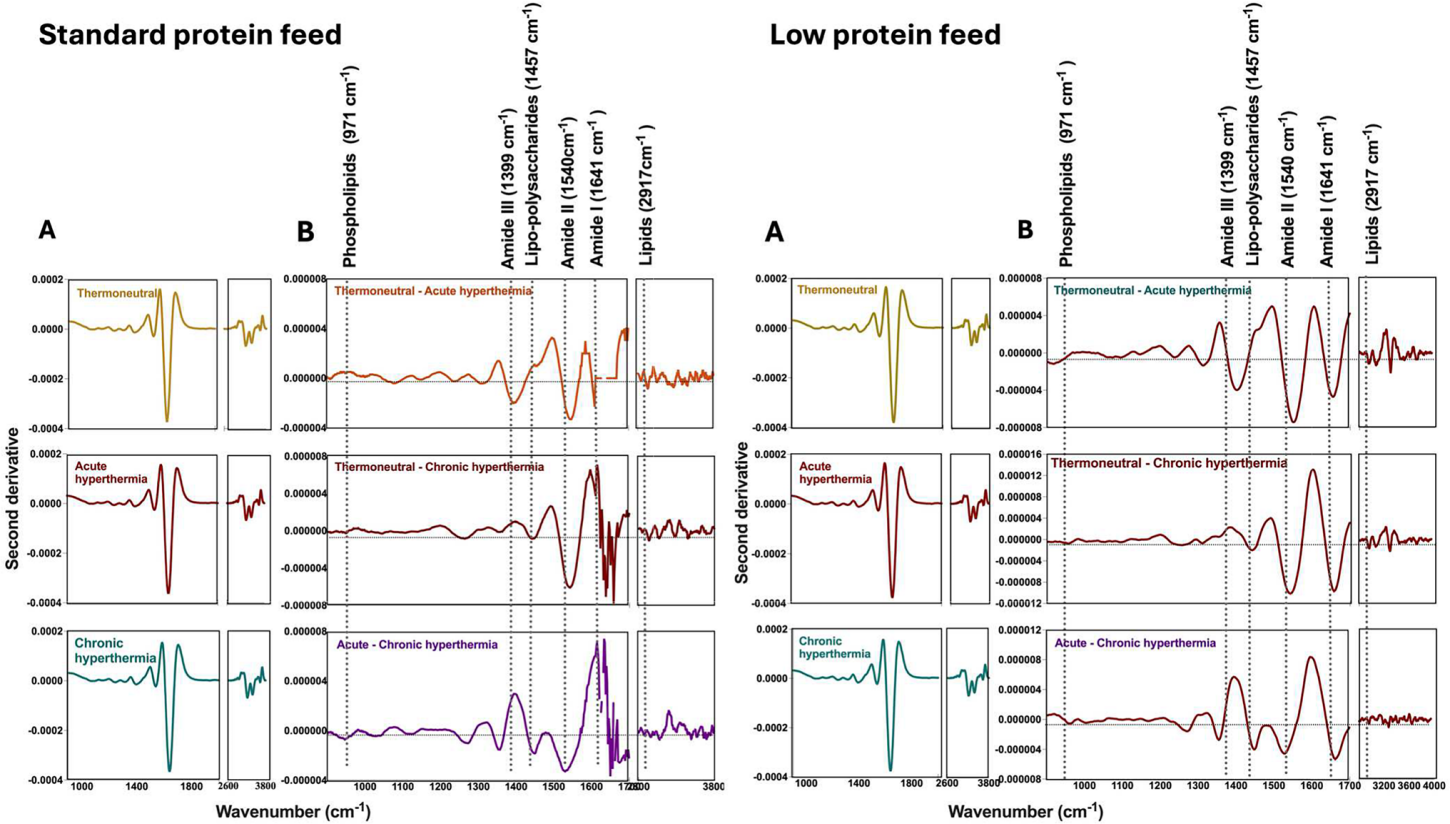
**A** Second derivative mid infrared (MIR) spectra of thermoneutral (TN), acute (AHS) and chronic (CHS) hyperthermia plasma from sows receiving a standard (left) or low crude protein diet (right). **B** Differences in the second derivative mid infrared spectra between the plasma samples. Top: normothermia-acute hyperthermia; Middle: normothermia-acute hyperthermia (AHS); Bottom: chronic hyperthermia (CHS) in both, standard (left) and low-level (right) of protein in the diet

**Fig. 4:**
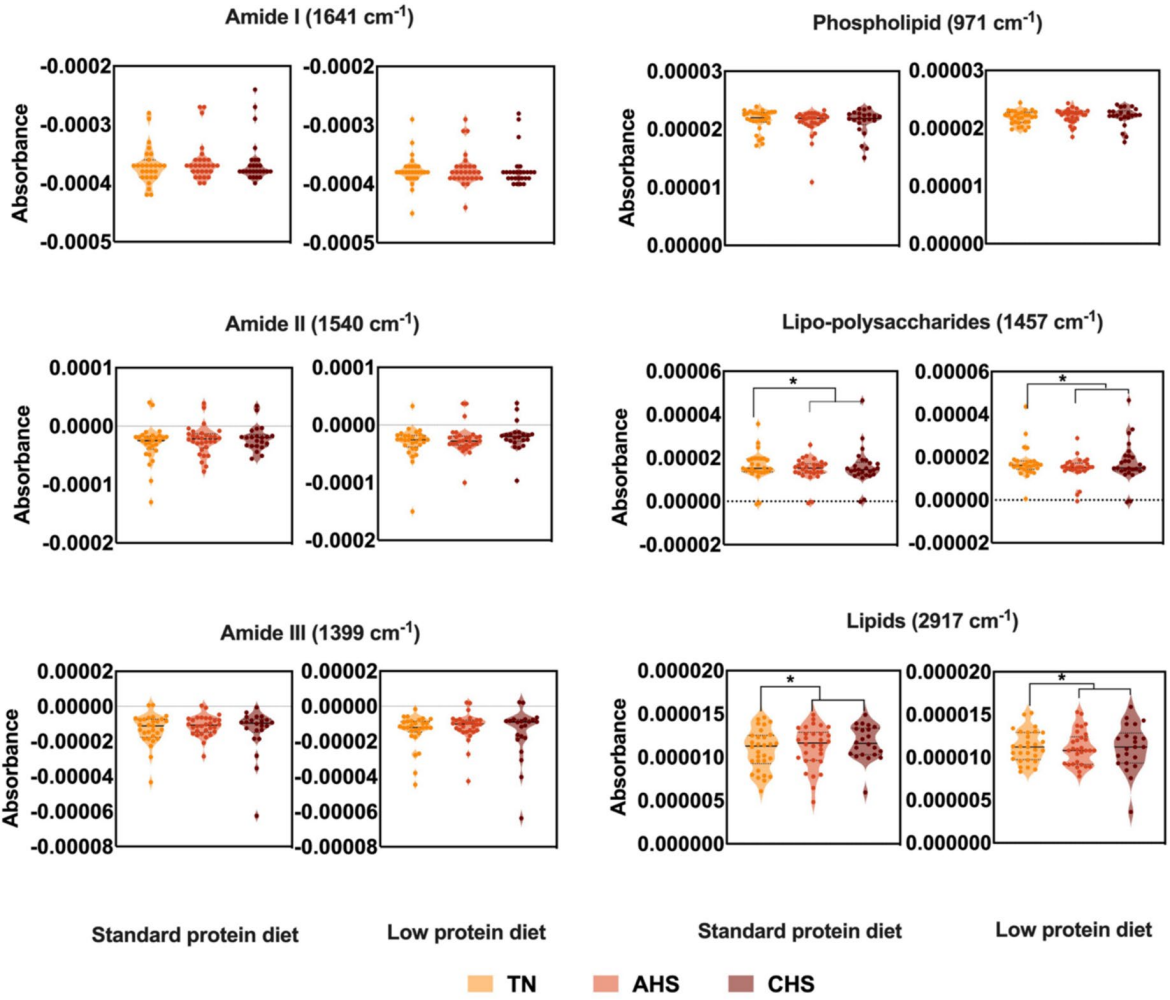
Absorbance values at specific wavenumbers corresponding to amide groups (I, II and III), phospholipid, lipo-polysaccharide, and lipid regions of the plasma samples from the three environment stress levels (TN, AHS and CHS) and two levels of protein (Standard -left- and Low -right-) analysed using mid infrared spectroscopy in the plasma of sows under thermoneutral or heat wave. An asterisk means a significant difference (*P* < 0.05)

Visual inspection of the average second derivative MIR spectra (Fig. 3, panels A) of the plasma samples collected from lactating sows under TN (top), in AHS (middle), or CHS (bottom) stress, fed either with a standard protein (20% CP) or a low protein (16% CP) diet appeared to be similar. To detect changes in the MIR spectra of the samples due to heat stress, averaged absorbance spectrum of the plasma samples in TN (normothermia) were subtracted from AHS and CHS groups and between AHS and CHS (Fig. 3, panel B top, middle, and low row, respectively). Subtle changes at the region around 971 cm^−1^, associated with the symmetric stretching modes of phosphate monoester in phosphorylated proteins (Yano et al. 2000; Baker et al. 2016; Talari et al. 2017), around 1635 cm^−1^, 1546 cm^−1^, and 1397 cm^−1^, associated with the stretching frequencies of N–H groups of amides I, II, and III bands of proteins, respectively (Barth 2007; Yang et al. 2015; Lorenz-Fonfria 2020). Changes in the MIR spectra around 1457 cm^−1^ were associated with the CH_2_ symmetric and asymmetric bending linked to lipo-polysaccharides, while around 2971 cm^−1^, attributed to CH_2_ and CH_3_ asymmetric stretching of lipids (Talari et al. 2017; Lin et al. 2000). In addition, the absorbance values of the plasma samples collected from animals under AHS were higher compared to the plasma from animals under TN and CHS in the wavenumbers associated with phospholipids and amide groups in both diets (Fig. 4). However, a significant increase in the absorbance values was observed between the plasma samples from TN and HS groups in the lipo-polysaccharides and lipid regions (*P* < 0.05) in both diets (Fig. 4) (Baker et al. 2016; Talari et al. 2017).

The second derivative MIR spectra of the plasma samples collected from the different treatments were used to develop classification models based on LDA. The plasma samples collected from both AHS and CHS groups were combined and identified as HS for classification purposes. The LDA classification results for the plasma samples are reported in the confusion matrix shown in Table 3. Overall, the plasma samples collected from animals under HS were correctly classified independently of the protein level in the diet (> 70%).

**Table 3:** Confusion matrix for the linear discriminant analysis of the plasma samples under thermoneutral (TN) or heat stress (HS) environments for all samples (both diets combined) and for each diet (standard

			Actual	
			TN	HS
Predicted	All samples (*n* = 162)	TN	39 (56%)	30
		HS	24	69 (74%)
	Standard protein samples (*n* = 81)	TN	17 (56%)	21
		HS	10	33 (76%)
	Low protein samples (*n* = 81)	TN	23 (60%)	15
		HS	13	36 (73%)

*N* number of samples, *TN* thermoneutral, *HS* heat stress. In brackets, percentual proportion of correctly classified samples

**Fig. 5:**
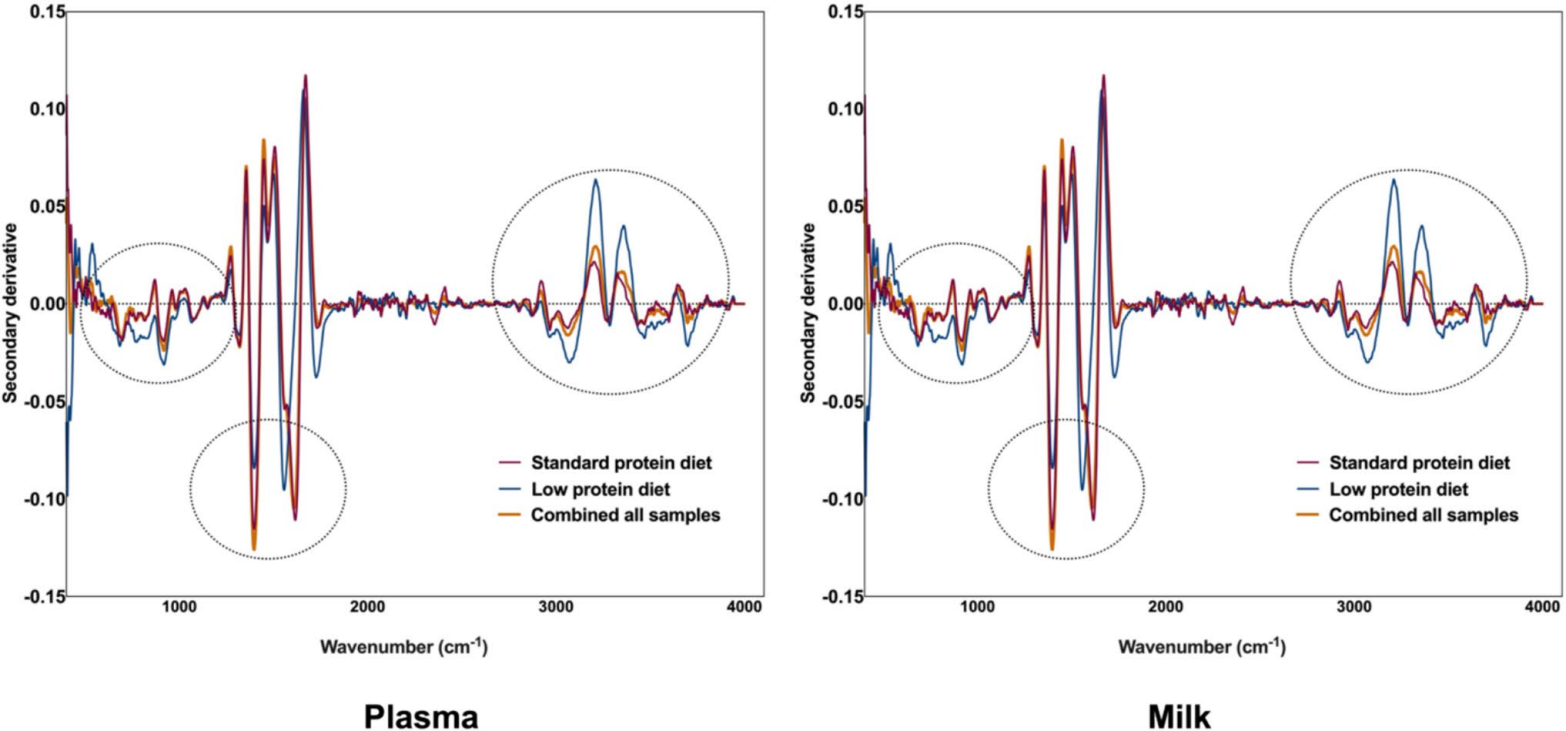
Optimal loadings used by the linear discriminant analysis model to classify plasma (left) and milk (right) samples from lactating sows under heat stress and two levels of protein (standard and low) analysed using mid infrared spectroscopy

To understand which wavenumbers contributed to explaining the LDA classification results, the loadings were interpreted. The highest loadings (Fig. 5, panel A) used to develop the LDA models to classify the plasma samples were observed around 3063 cm^−1^ corresponding to C-H groups associated with lipids and unsaturated fatty acids (Talari et al. 2017), around 2966 cm^−1^ (stretching C-H) corresponding with fatty acids and lipids, and around 1746 cm^−1^ corresponding to C = O of esters groups and fatty acids (Baker et al. 2016; Talari et al. 2017). Three loadings were also observed around 1604 cm^−1^, 1395 cm^−1^, and 1317 cm^−1^ associated with amide groups (amide I, II, and III) of proteins (Barth 2007; Yang et al. 2015; Lorenz-Fonfria 2020). Loadings were also observed around 1072 cm^−1^ associated with either carbohydrates or phosphate stretching modes of C-O, phosphodiester groups from DNA in disordered structure, and around 913 cm^−1^ associated with phosphodiester stretching bands from RNA and DNA (Baker et al. 2016; Talari et al. 2017).

### Interpretation of the MIR spectra of milk samples

**Fig. 6:**
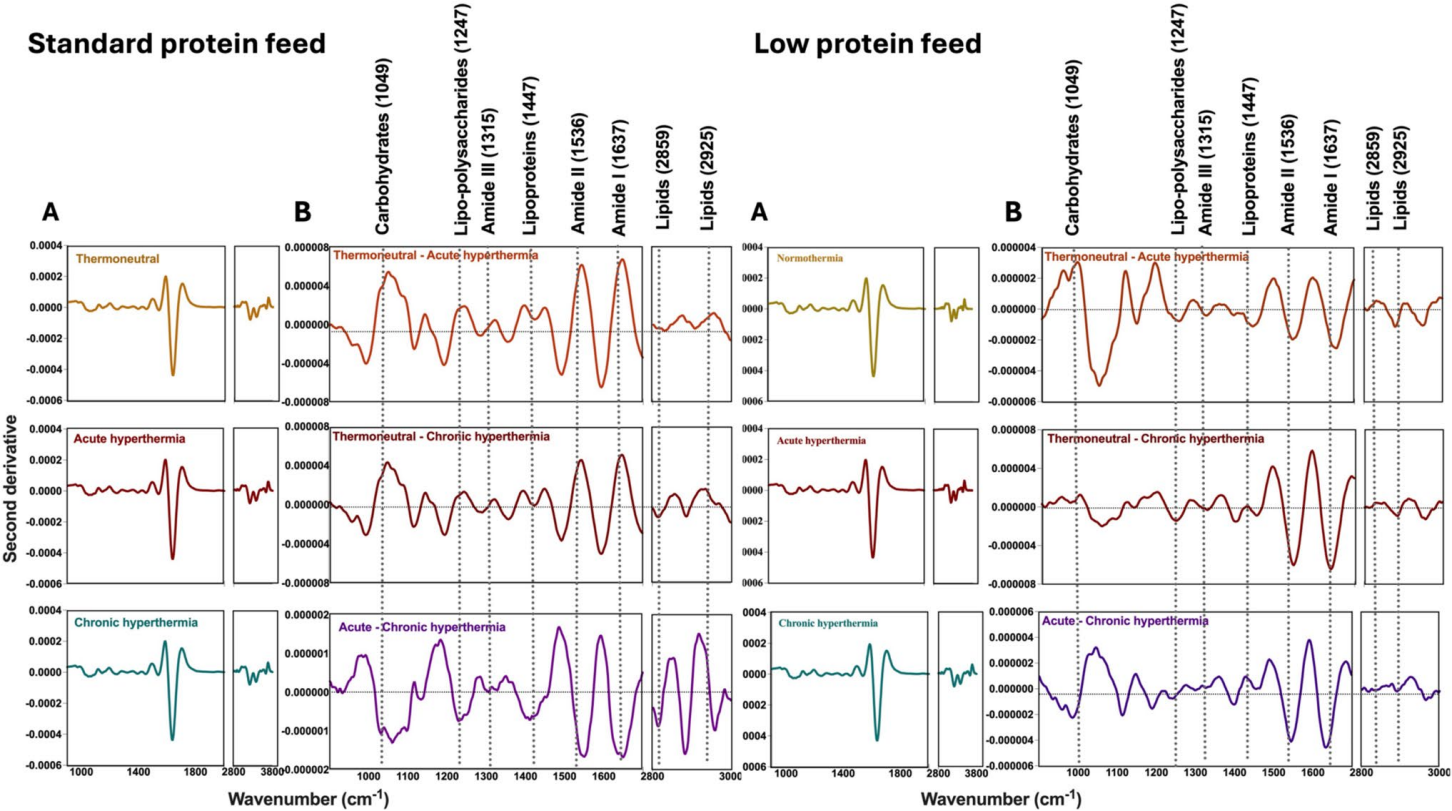
**A** Second derivative mid infrared (MIR) spectra of thermoneutral (TN), acute (AHS) and chronic (CHS) hyperthermia plasma from sows receiving a diet with standard or reduced level of protein. **B** Differences in the second derivative mid infrared spectra between the milk samples. (Top: normothermia-acute hyperthermia; Middle: normothermia-chronic acute hyperthermia (AHS); Bottom: acute-chronic hyperthermia (CHS) in both, standard and low-levels of protein in the diet

**Fig. 7:**
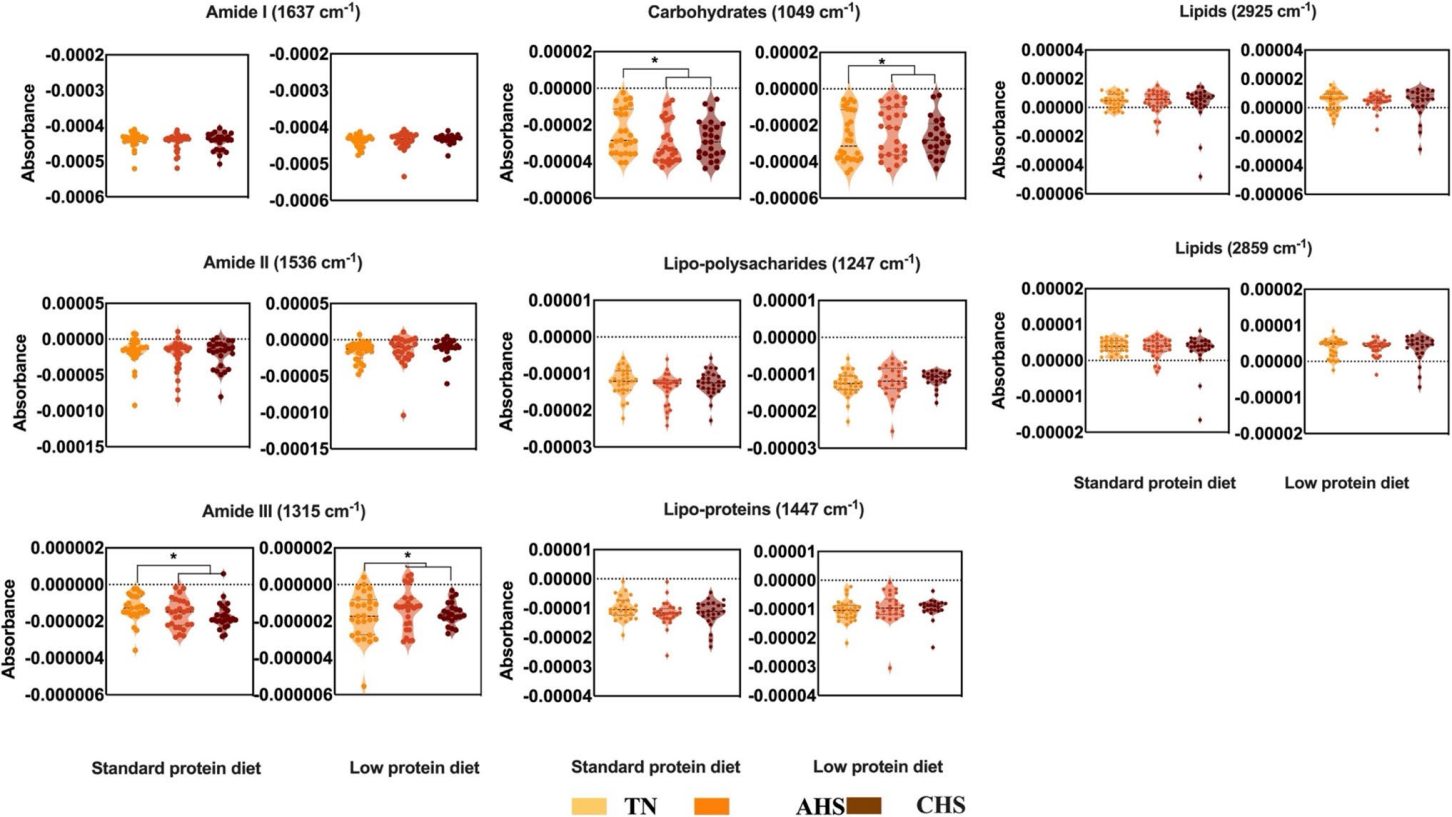
Absorbance values at specific wavenumbers corresponding to amide groups (I, II and III), carbohydrates, lipo-polysaccharide, lipo-proteins, and lipid regions of the plasma samples from the three environment stress levels (TN, AHS and CHS) and two levels of protein (Standard -left- and Low -right-) analysed using mid infrared spectroscopy in the milk of sows under thermoneutral or heat wave. An asterisk means a significant difference (*P* < 0.05)

Figure 6 panel A reports the second derivative of the mean MIR spectra of the milk samples collected from lactating sows under TN (top), in AHS (middle) or CHS (bottom), fed either with a standard protein (20% CP) or a low protein (16% CP) diet. Figure 6 (Panel B) reports the differences between the averaged spectrum samples collected under TN conditions, AHS and CHS groups and between AHS and CHS (top, middle, and low row, respectively). As observed in the plasma samples, the averaged MIR spectra of the milk samples from TN were subtracted from the averaged MIR spectra of sows in the HS group. Changes in the MIR spectra of the milk samples were observed around 1049 cm^−1^, which was associated with carbohydrates and in the lipo-polysaccharides region (1247 cm^−1^). In addition, changes were observed around the amides I, II, and III regions (1635, 1546, and 1315 cm^−1^ respectively), lipo-proteins (1447 cm^−1^), and lipids (2859 cm^−1^ and 2925 cm^−1^) (Barth 2007; Yang et al. 2015; Lorenz-Fonfria 2020). The statistical analysis of specific wavenumbers indicated significantly differences in the absorbance values (*P* < *0.05*) assigned to the carbohydrates and amide III regions (Fig. 7) in the milk samples of animals from the HS treatment compared with animals from the TN group, independently of the levels of protein in the diet.

The MIR spectra of milk samples from the different treatments were also classified according to HS using LDA (see Table 4). Like the results obtained for plasma, a high percent of correct classification was obtained for the milk samples across treatments. The milk samples collected from the animals under HS were correctly classified above 70%, while milk collected from animals under TN was less than 50% correctly classified in both standard and low protein diets. The highest loadings (Fig. 5, panel B) used to develop the LDA classification models were observed in wavenumbers associated with carbohydrate and polysaccharides between 1400 to 900 cm^−1^ (Baker et al. 2016; Talari et al. 2017). High loadings were also observed around 1072 cm^−1^ and 913 cm^−1^ corresponding to the same assignation described in the section above (Baker et al. 2016; Talari et al. 2017).

**Table 4:** Confusion matrix for the linear discriminant analysis of the milk samples collected under thermoneutral (TN) or heat stress (HS) environments

			Actual	
			TN	HS
Predicted	All samples (*n* = 155)	TN	32 (46%)	42
		HS	23	58 (71%)
	Standard protein samples (*n* = 79)	TN	17 (46%)	20
		HS	10	32 (75%)
	Low protein samples (*n* = 76)	TN	16 (45%)	20
		HS	12	28 (70%)

*N* number of samples, *TN* thermoneutral, *HS* heat stress. In brackets, percentual proportion of correctly classified samples

## Discussion

This study observed changes in the MIR spectra of plasma and milk samples collected from sows under TN conditions compared with HS. Specific changes in wavenumbers associated with biomarkers or metabolites related to lipo-polysaccharides, amide groups of proteins, and lipids were also observed, independently of the diet consumed by the sow. Changes in some of these wavenumbers have also been reported when the plasma of rats under HS was analysed using MIR spectroscopy (Lin et al. 2000), or biomarkers were predicted in the milk of dairy cows (Giannuzzi et al. 2023).

The HS is considered one of the main environmentally imposed stressors changing animal homeostasis. In this study, the reduction of feed is considered a protective and adaptive mechanism employed by the animal to counteract the increase in metabolic heat production. However, reduction of intake can create a negative energy balance, inducing both lipolysis and proteolysis in pigs (He et al. 2019; Gonzalez-Rivas et al. 2020). Under the HS wave, the body temperature of the sows rose significantly above normal levels. It kept higher even during the night period, where the temperature slightly dropped, although was not dissipated to reach normal levels. Hyperthermia could induce changes in internal temperature regulation and activation of the inflammatory and endocrine stress response (Török et al. 2014; Kultz 2005; Zhao et al. 2022). Persistent hyperventilation due to panting during HS produces alkalosis, a condition where blood becomes too alkaline due to the excessive loss of carbon dioxide through rapid breathing. Imbalanced blood pH can impair metabolic processes, membrane integrity and protein structure (Gonzalez-Rivas et al. 2020). Respiratory alkalosis, hyperthermia and hypoxia are stressors altering the gut protective epithelial ultrastructure and permeability in pigs, inducing changes in the microbiota profile (Hu et al. 2022; Cantet et al. 2021; Mayorga et al. 2020). These changes have been associated with the translocation of resident microflora and endotoxins, such as lipo-polysaccharides, to the systemic circulation in heat-stressed animals (Wang et al. 2020; Pearce et al. 2013; Mayorga et al. 2020). In addition, HS can have non-specific effects on the integrity of the cell, eliciting membrane deformation, enzyme inactivation, increased protein misfolding, production of oxidative stress, and release of proinflammatory mediators while triggering the activation of defence mechanisms such as heat shock proteins (Ringseis and Eder 2022; Lian et al. 2021; Lin et al. 2020; Slimen et al. 2016) or determining the accumulation of abnormal proteins (Kultz 2005; Balogh et al. 2013; Cherkasow et al. 2013; Török et al. 2014; Cui et al. 2021). Changes in the lipid profile of both plasma and milk due to hyperthermia have been linked to energy storage and lipid membrane stability (He et al. 2019; Balogh et al. 2013). For example, in pigs with high energetic demands (e.g. gestation, lactation), HS increases lipid catabolism not only by increasing the concentration of non-esterified fatty acids in plasma (released from adipose tissue due to the action of the somatotropin) but also by enhancing the glycerolipid pathway to supply more energy from glycerol metabolism (He et al. 2019). In this study, it can be speculated that similar effects might explain the changes in the absorbance values at specific wavenumbers in the MIR region (e.g. lipo-polysaccharides, amide, lipids) in both plasma and milk samples analysed. The HS impact on cellular components might alsobe explained by the changes in the MIR spectra in wavenumbers associated with amides and lipids. Additionally, changes in cellular components can be indirectly associated with the observed differences in amides I and II, which might relate to structural changes in proteins, while amide III it is associated with peptides, DNA, and RNA (Talari et al. 2017). HS has also been related to alterations in the secondary structure of the proteins (e.g. increased beta-sheet content and a decrease in alpha-helix). These changes can be observed in the wavenumbers associated with Amide I, II and II bands (Ausili 2023). These wavenumbers have been described as applicable for studying heat-induced protein denaturation and conformational changes (Wolkers and Oldenhof 2010). Gianuzzi and collaborators (2023) also reported that milk proteins, particularly those associated with inflammatory processes can be detected in the amide band.

## Conclusions

The results indicated that MIR spectroscopy, combined with chemometrics, could identify changes in the spectra associated with heat stress in wavenumbers corresponding with amide groups (proteins), lipids, lipo-polysaccharides and carbohydrates. This study showed the potential of MIR spectroscopy to deliver cost-effective, rapid screening and diagnostic tools to predict and monitor biologically relevant changes in animal physiology with high potential for palliative strategies such as specific diet formulations. Although the results of this study require further validation, this study has shown that MIR spectroscopy might allow for the development of high-throughput methods to monitor metabolic changes in animals due to HS. Furthermore, using MIR spectroscopy will allow for the development of calibrations to quantify biomarkers associated with HS. This, in turn, highlights potential applications relevant to animal welfare, adaptability to climate change, or the advent of disease outbreaks significant for intensive production systems.

## Data Availability

None of the data were deposited in an official repository. The data that support the study findings are available upon request.
